# Towards A Self Adaptive System for Social Wellness

**DOI:** 10.3390/s16040531

**Published:** 2016-04-13

**Authors:** Asad Masood Khattak, Wajahat Ali Khan, Zeeshan Pervez, Farkhund Iqbal, Sungyoung Lee

**Affiliations:** 1 College of Technological Innovation, Zayed University, 144-534 Abu Dhabi, UAE; farkhund.iqbal@zu.ac.ae; 2 Department of Computer Engineering, Kyung Hee University, Yongin-Si 446-701, Korea; wajahat.alikhan@oslab.khu.ac.kr (W.A.K.); sylee@oslab.khu.ac.kr (S.L.); 3 School of Engineering and Computing, University of the West of Scotland, Paisley, PA12BE, Scotland, UK; zeeshan.pervez@uws.ac.uk

**Keywords:** activity recognition, change management, u-healthcare, decision support system, service recommendation

## Abstract

Advancements in science and technology have highlighted the importance of robust healthcare services, lifestyle services and personalized recommendations. For this purpose patient daily life activity recognition, profile information, and patient personal experience are required. In this research work we focus on the improvement in general health and life status of the elderly through the use of an innovative services to align dietary intake with daily life and health activity information. Dynamic provisioning of personalized healthcare and life-care services are based on the patient daily life activities recognized using smart phone. To achieve this, an ontology-based approach is proposed, where all the daily life activities and patient profile information are modeled in ontology. Then the semantic context is exploited with an inference mechanism that enables fine-grained situation analysis for personalized service recommendations. A generic system architecture is proposed that facilitates context information storage and exchange, profile information, and the newly recognized activities. The system exploits the patient’s situation using semantic inference and provides recommendations for appropriate nutrition and activity related services. The proposed system is extensively evaluated for the claims and for its dynamic nature. The experimental results are very encouraging and have shown better accuracy than the existing system. The proposed system has also performed better in terms of the system support for a dynamic knowledge-base and the personalized recommendations.

## 1. Introduction

It is a well-recognized fact that the effects of ageing are causing huge social and economic challenges. At the same time the rise in living standards with the advancement of new technology also has raised the importance of sophisticated healthcare applications and services with low cost. These are the challenges which are currently affecting all countries within Europe and are having an impact on a wide range of policy domains. The European Commission recognized that by 2025, more than 20% of Europeans will be 65 or over. The diversity of needs of the elderly in conjunction with the requirement to offer both financially viable and sustainable ageing supporting paradigms have shown a lack of compelling solutions which have had widespread uptake. To facilitate healthcare and reduce the cost associated with the healthcare applications, systems, and services, the U.S. federal government is investing $27 billion in health information technology [1].

According to the World Health Organization it has been reported that no group can benefit more than the elderly from regularly performed exercise [2]. From a healthcare perspective the positive benefits of exercise have been associated with the detection, prevention and treatment of many chronic conditions, for example hypertension, diabetes, Parkinson’s, heart disease and even cognitive decline [3]. In addition to regular exercise, the effects of maintaining a healthier diet have also been recognized [3,4]. Nevertheless, although there is appreciation of the need for such a diet, there is a lack of evidence that this has actually had an impact on the improvement of dietary management. One explanation for this may lie in the fact that there is a general lack of nutritional knowledge within the population. It has recently been stated that the use of pervasive technologies could facilitate and promote healthier food preparation [4]. In addition to exercise and nutrition, a third component can be considered within this wellness paradigm, namely social integration. According to the World Health Organization’s Active Ageing program, active ageing highlights the importance of health [5].

The rapid growth and increasing usage of data is the main reason for higher complexity of the data collected in the specified field of study* i.e.*, healthcare. The complex data also introduce uncertainty, specifically relating to the problems in healthcare services [6,7]. Nowadays, people are more interested in better healthcare, which results in high cost healthcare services. The challenge is cost effective quality healthcare services availability [8]. Various wireless technologies are in use to provide better healthcare services. In [9], the authors proposed an approach with Electronic Health Record (EHR) for existing hospital systems. The rule engine and the rule-base for the guidelines are integrated. With static rules, their main drawback is strict modeling of information according to the input types for different components. CodeBlue [10] is a technology developed at the Harvard Sensor Network Lab that supports physicians and nurses in monitoring patients. Research on reminder systems for elders is gaining importance. These are mostly plan-based approaches to decide when and how to prompt subjects effectively [11]. In addition, a location-based reminder system was introduced in [12]. However, context for reminders is more important than just location or time. HYCARE [13] is a recent reminder system that consider context, develops a schedule for various reminder services, and remedies the possible conflicts. In [14], the system uses an ontology to incorporate context for intelligent processing and recommendations based on the processed context. However, the system is more of a homecare system than a healthcare system.

To extract useful information [15], researchers from mathematics, medical and computer science research areas have worked on a number of approaches [16,17] that support building expert systems. However, these theories are restricted to limited domains where they can perform better. One system [18] is the most recent that is based on ontology for recommendation services and incorporates rules for manipulating the recognized activities. In [19], the authors focused on real-time activities recognized using diverse sensors performed by patients and then use these activities and domain knowledge represented in ontology for situation analysis with the help of experts designed rules. In the healthcare domain, rule-based systems are preferred over machine learning based systems [20,21]. Medical doctors can better interact with systems using rule-based approaches and can compile new rules. On the other hand [6] focused on the social interaction of patients. Based on the experience shared by the patient and patient’s community of the same disease type, the system generates intelligent service recommendations and eliminates the isolation of the patients. The authors in [22] presented a real time application deployed at an ICU, connected to a microbiology lab and a patient management system. The application monitors a patient on a daily basis for their daily behavior and symptoms.

The existing systems are based on one input source (one sensor) and in some cases use imperfect information [23] for service recommendations. The existing systems do not consider the integration of activity information with patient profile information and patient nutrition intakes for service recommendation. The existing systems are also static in nature and are not adaptive to the dynamic nature of the patient’s behavior and the environment. To facilitate achieving the objective of dynamic decision and service suggestions for diet based on patient activities and nutrition intake, the proposed approach of Social Wellness for Elderly (SWELL) is presented in this research. It is based around the improvement in general health status of the elderly through the use of an innovative service to align dietary intake with daily activities, promoted through a social network using contemporary Web 2.0 and semantic web technologies. An underlying social network will provide the backbone for this innovative service and ensure that feedback, perhaps driven by a self-management paradigm, will provide recommendations for appropriate nutrition intake and physical activities.

The focus of this research work is on the Dynamic Decision Support System (DDSS) component of the SWELL [8], whereas the SWELL architecture is briefly discussed in the next section. This research is an extension of our previous publication [8]. The extensions are focused on the evolutionary aspects of SWELL and the methodology followed to achieve the results. Cloud- based DDSS actively listens to its outer world agent (a smartphone) for data that include user daily life activities and user daily life nutrition intake. DDSS uses this information for appropriate recommendations on physical activities and nutrition intake. Any change in user behavior detected using activity recognition or changes suggested by a caregiver will automatically be updated; the next recommendation will be based on the changed user preferences and caregiver’s suggestions. The Knowledge Repository (KR) is an ontology where patient information are formally represented in an ontology. Any change in user behavior and change suggestions from caregivers are updated dynamically in the KR. DDSS has been tested for its recommendations and dynamic recommendations against an existing system, and has proved better performance. The dynamic KR and its services for changes in repository and rules have also been implemented and tested extensively. The experimental results are very encouraging for robust service recommendations and dynamic change management. The reported results are mostly on the proposed system working for change management, evolving nature and response time, whereas the proposed system working for aspects on healthcare are mainly reported in [8].

This research paper is arranged as follows: Section 2 describes a detailed view of SWELL and its different components with their functionality. Section 3 presents the proposed system in detail. Section 4 discusses the experimental results. Finally we conclude our discussion in Section 5 and talk about future directions.

## 2. Social Wellness for the Elderly

**Figure 1:**
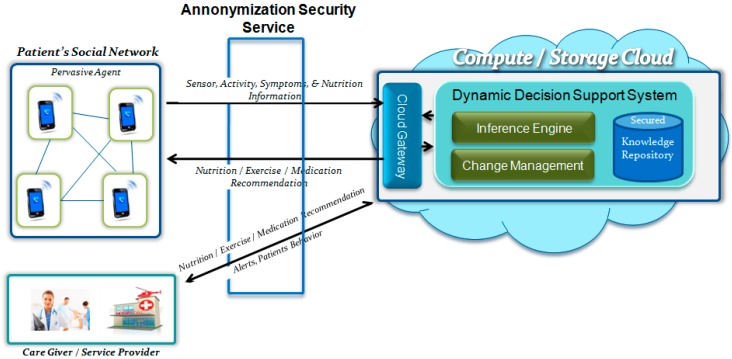
The component wise architecture of SWELL showing the information flow and processing at different levels.

In this section we describe the component-wise architecture and working model of SWELL. Though it is not the focus of this paper; however, it is important to explain the overall working model of SWELL for a better understanding of the proposed DDSS. SWELL is basically composed of three main components (see Figure 1),* i.e.*, (1) Pervasive Agent (PA) to collect data; (2) Secure Cloud Storage (SCS) to securely store the collected data; and (3) Dynamic Decision Support System (DDSS) to generate services. DDSS is the focus of this research paper, so in this section the focus will be on PA and SCS.

The PA (smartphone) is the outer world agent of the SWELL system that is used by the patients during their daily life. While it is in use, it provides the facility of recognizing the user location, activities, and vital health data with the help of embedded sensors and with the wirelessly connected medical devices. Nine different sensors of the PA are used to recognize the patient activity: Global Positioning System (GPS), Accelerometer, Camera, Proximity, Light, Time, Gyroscope, Bluetooth, and Microphone. The patient activities detected using these sensors, the nutrition intakes captured using a camera on the PA, the social interaction using Short Message Service (SMS) or community chat on the PA, and ElectroCardioGram (ECG), Heart Rate, Blood Pressure, and Glucose Level data that is collected using the PA are all forwarded to the DDSS hosted on the Cloud server. After intelligent manipulation on the DDSS, the DDSS generates personalized recommendations for nutrition intake, physical activities (exercise), and future healthcare suggestions and medication recommendations. These recommendations are provided to the patients on their PAs. For details of the workings of PA readers may refer to [24].

From a social interaction perspective the objective of SWELL is to improve quality of life and eliminate the social isolation. This is achieved through promoting improvement in both nutrition intakes and activity related activities, coupled with improved social interactions with family, friends, and professional healthcare providers. SWELL engages all the stakeholders and promotes social sharing. Patients share their activity and health information with other stakeholders (*i.e.*, patients and medical doctors) in their social graph. The granularity of the shared information will be controlled by the participants themselves while setting the privacy features provided in SWELL. The Secure Cloud Storage (SCS) achieves data privacy while supporting seamless data sharing among the involved users. SCS works as a secure bridge between the data in the cloud and the users who want to access it. Its main responsibility is to ensure that the access control policies are intact and legitimate users always accesses the required data. To achieve this, information about legitimate users is maintained, and digital certificates along with identity assertions are used to access the data while preserving privacy. The current version is on private compute (cloud) server and once all the components are tested for workability and reliability then will export it to public cloud server. To support the design and deployment, overall methodology has already been designed, implemented, test and published in [25].

## 3. Dynamic Decision Support System

**Figure 2:**
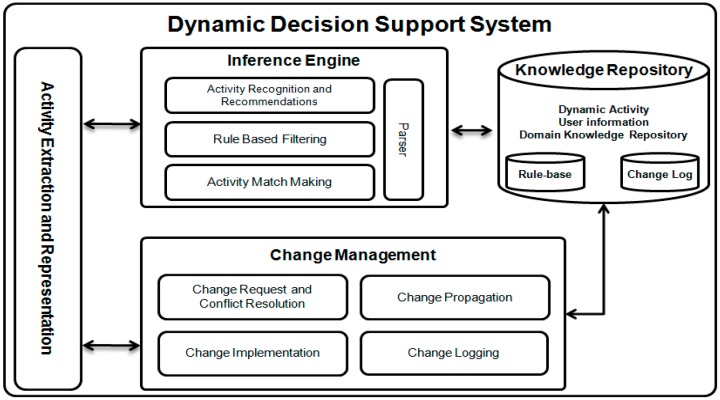
Dynamic Decision Support System (DDSS) architecture for information inflow, processing, and recommendation outflow.

Today’s healthcare and lifecare systems are widely used for better, timely, and low cost healthcare services and recommendations. Most of these are based on the concept of activity recognition and also using expert knowledge as guidelines to produce recommendations based on patient situation. In this research work we focused on real time robust activity and nutrition intake information manipulation for generating intelligent personalized physical activities, medication, and nutrition intake recommendations, where this is all achieved with the Dynamic Decision Support System (DDSS). This section provides details on the DDSS workings (see Figure 2), its information flow, storage, manipulation, and recommendation generation. DDSS takes as its input activities and nutrition intake communicates with the outer world agent,* i.e.*, PA, and also produces the results (recommendations) back to the PA. DDSS is composed of three main components discussed below.

### 3.1. Knowledge Repository

**Figure 3:**
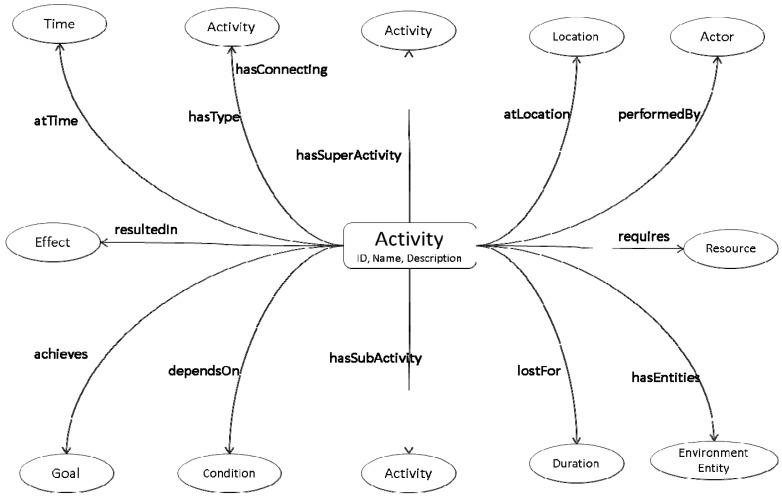
Activity, actor, and effects relationship modeling in ontology for representation of information in Knowledge Repository.

The Knowledge Repository (KR) serves as the backbone of DDSS. It contains the evolving activity information, social interaction, patient’s profile information, domain knowledge, and expert knowledge on nutrition intake. Based on this information and newly detected activities, the Inference Engine generates recommendations. The information in the KR needs to be represented in a format that is semantically rich and formally structured. For this purpose we have modeled the information using an ontology. The knowledge in the KR is semantically linked (see Figure 3) for later processing using an Inference Engine (IE). The following axiom represent the proposed abstract model for representing knowledge in KR ontology represented by *O*. Any activity (physical) performed by user or any interest and post (cyber activity) shared by subject on social media is considered as user performed activity which is then connected to related nature of it. These activities are independent for their existence; however, most of the time dependent on subject’s preference. At the same time there are also some elements, such as activity effects, consequent actions, duration of activity and location where activity was performed. Proper linkage, storage and management of all this information is necessary for better situation analysis and recommendations. The following ontology is developed to handle all such aspects of activities and their corresponding elements.

*O* ≡ ∃Activity.(DetectedActivity ⊔ SocialInteraction) ⊓ Activity.relatesTo(∃Nutrition) ⊓ ∀Activity.hasPerformer(1Actor.has(Preferences)) ⊓ ∃Activity.has(Activity ⊔ Effects ⊔ Actions ⊔ Location ⊔ Duration ⊔ Condition)

Once the inference part is completed from the information including activity information, profile information, and nutrition intake used from KR and PA, then the inferred knowledge is filtered using the expert (nutritionist) knowledge encoded in rules and stored in the rule-base that helps to generate personalized recommendations for the patients for their daily life physical activities, medication, and nutrients intake. Both the Dynamic Activity Repository (DAR) and rule-base evolve as the system learns more about the patients or environment or the experts introduce more knowledge.

**Figure 4:**
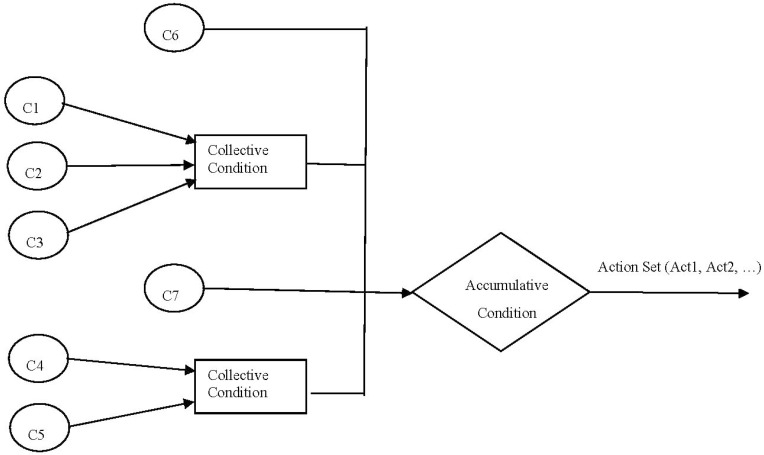
Abstract structure of a rule. Set of optional and mandatory conditions, confidence building, and resultant action.

Recommendations are mainly based on reliable collected facts and intelligent interpretation of these facts. Facts represent knowledge about the situation at hand and rules represent relationships among the facts. Based on these rules, the Inference Engine generates recommendations. Rules are mainly used to get end results in optional or mandatory combinations. For example, Figure 4 shows the consequent effects of a combination of optional and mandatory facts (C_1_….C_7_) that builds confidence for a rule in a given situation and results in a consequent action. The action can be a single action or can be a set of actions resulting from a particular rule.

**Figure 5:**
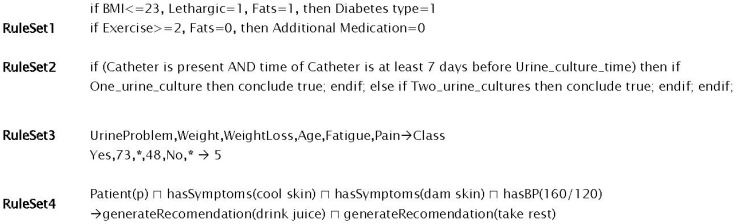
Examples of procedural (if…then) rules, rule in arden syntax (only logic part), comma separated rule used by Rough Set, and description logic rule.

All rules have two main components,* i.e.*, condition and action, that makes them easy to model. The representation of condition and action components in different rules is also different (see Figure 5). In Figure 5, the RuleSet1 represent procedural rules, the condition and action components are visible with the help of *if* and *then* constructs (where BMI is Body Mass Index). Arden syntax in RuleSet2 of Figure 5 also uses *if* and *then* constructs; however, they import the definition of these construct from the Data and Action segment of the rule where a rule itself is defined in the Logic segment [26]. Conditions in the rules (RuleSet3 of Figure 5) of Rough Set (lower and upper set based approximation scheme for reasoning and mining) based rule-base are separated using comma and action is targeted using the arrow. In RuleSet3 of Figure 5, a description logic rule is given where the action is also targeted by an arrow that is the consequent effects of conditions combined together using different logical operators as antecedent.

**Figure 6:**
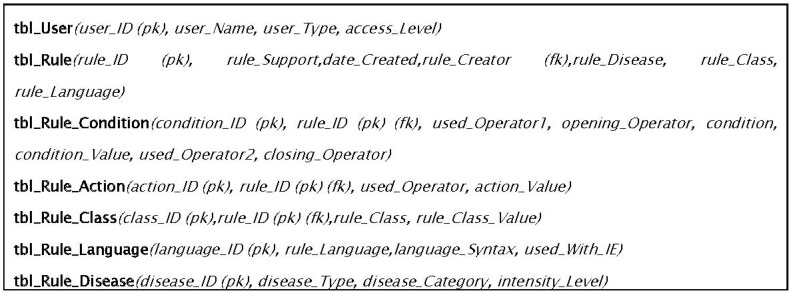
Generic storage structure for rules storage in Rule-base.

With different rules representational formats, using different operators (logical, mathematical, and relational) operators makes a single storage structure design a bit tough. To handle this, we have developed a relational schema (see Figure 6) and model a rule as an entity. The condition and action parts are separate and is connected by distributing its primary key (identifier) in their respective table and as a foreign key (referencing/relative identifier) as shown in Figure 6.

In addition to a storage repository for activity information and rule storage, we also need to store the changes applied to KR and Rule-base to support the dynamic nature of the proposed system. To store all the changes applied and suggested we have modeled the Change Log using an ontology to capture all the semantics of the changes and their dependency on other entities. For detail of the structure and change management of the changes applied, please refer to our previous work on ontology change management [27].

### 3.2. Activity Extraction and Representation

**Figure 7:**
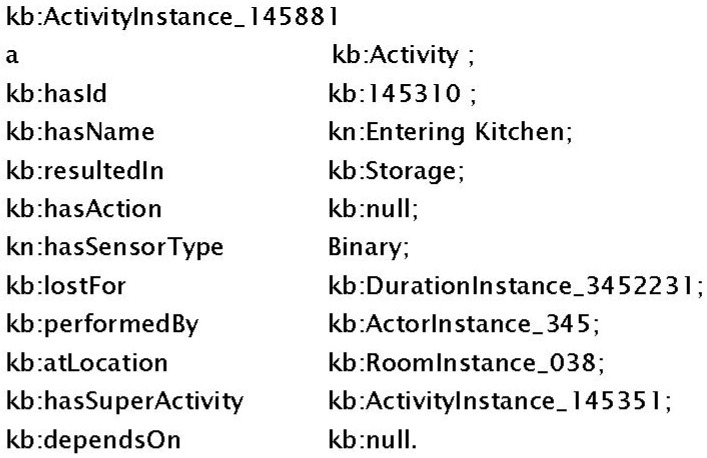
Ontological representation of activity in N3 notation.

This module is the interaction point of the DDSS to the outer world agent PA. It accepts all the data and communication from the PA and then models it using the KR structure. It is also responsible for sending the response back to the PA. Any communication in this module is termed an activity; even social interaction is also termed an activity. The interaction and the activities are all stored in the KR. Any new patient information or suggestions/recommendations/medications from caregiver also passes through this module. Once the activity is extracted then it is represented (see Figure 7) in the ontological structure (using N3 notations) that is proposed in this system for representation and storage of information in the KR. The representation shows that an activity is performed (in this case it is Entering Kitchen) which will result in only storage of this activity in KR with no consequent action due to which connected action is set to null. Additional information with the activity, such as who performed the activity, where it is performed, for how long the activity is performed, how is the activity detected and what is super activity (if any) of this activity is also represented in Figure 7. This complete information is then logged in KR for later use. If it is related to any change in the existing knowledge then it is given to the Change Management (CM) module. If it is a newly reported activity by the PA then it is passed to Inference Engine for storage and recommendations generation.

### 3.3. Inference Engine

The Inference Engine (IE) is the brain of the DDSS. It is mainly dedicated to reasoning and to generating services. It involves KR for situation analysis and the Rule-base is used for recommendation generation. Newly detected activities are matched with knowledge from the KR. Here a match making process is initiated to find matching knowledge for the newly collected emerging information. Then the Rule-base is used to filter the reasoned (matched) knowledge and to make up to date and appropriate recommendations for social interactions, nutrition intake, and physical activities, such as rest and exercise. A two phase algorithm (see Algorithm 1) has been devised for the inference engine which are matched with the ontological information contained in KR and rule-based filtering based on the expert compiled rules contained in the Rule-base. Activity *A* is an activity detected and if it is a valid activity then it is assigned as member of ontology *O* in *Activity *class which is established earlier in Section 3.1. For valid detected activity, process of match making (Semantic Affinity) is initialized to find the semantic relatedness of activities detected and already stored. This relatedness will help in generating Higher Level Activity (HA) where their higher level activities can have consequent actions/recommendations and can also have only storage needs for later use as depicted in Algorithm 1. Here ontology *O* is not actually overwritten but in fact it is updated with new incoming information detected and received in the system.

**Algorithm 1.** Algorithm for activity information collection, manipulation, situation analysis, and recommendation generation.***Algorithm RecommendationGen (Process Patient’s Activities):***This algorithm predefines the matching threshold of activity to *ψ =* 0.70 with another activity.**Input:** Dynamic Activity ontology *O* contains domain knowledge and the patient’s profile information. Instance of newly detected activity *A*, *A*∈ *O. *Decision rules *R, *used for decision making.**Output:** Nutrition intake, social interaction, and Activity recommendations.**1.** /* Receive detected activities from Personal Agent */**2.**
*A* ← getActivity(*A*)**3.** /* Parse ontology for the knowledge, calculate semantic affinity (*i.e.*, semantic relatedness) */**4.**
*O* ← parse(*O*)**5.**
*O*_sub_ ← semanticAffinity(*A, O, ψ*)**6.** /* Read rules from the Rule Base maintained in an array R*/**7.**
*R[]*← readRules(Rulesbase)**8.** /* Start recommendation process using forward chaining */**9.**
**foreach**
*R_∆_* ∈ *R[]*
**do**
**10.**  /* Process for recommendation generation */**11.**  **If**
*R_∆_* : *A* ⊓ ∃ *O*_sub_.Activity ⊓ ∃ *O*_sub_.Action **then**
**12.**  /* Store the recommendations in an array */**13.**  RecomArr*[]* ← {x ∣ < *R_∆_*, x > Action}**14.**  ** else **
**15.**  /* Process for higher level activity */**16.**  *HA[]* ←*O* ⊓ {x ∣ < *A*, x > hasAssociation}**17.**  ** EndIf****18.** **EndLoop****19.** /* Execute valued recommendations and store higher activities*/**20.** execute(RecomArr*[ψ]*)**21.** *O^/^* ← *O + HA[ψ] ***22.** **End**

### 3.4. Change Management

CM is the main component that facilitates the dynamic nature of the proposed DDSS. The CM is responsible for KR and Rule-base evolution with the advancement of domain knowledge, modifications from experts, changes in patient behavior, social interaction and patient experience. CM accepts requests for new information additions as well as changes in rules from experts and automatic learning, then it implements the change request and propagates the changes to dependent components to minimize their effects. The associations and restrictions of ontology based knowledge-bases allow us to manipulate them in relative context to our needs. However, if the manipulation is in a manner which violate these restrictions then the ontology will result in invalid due to conflicts in it based on the restrictions. The conflicting resource can have cascading effects on other connected resources due to the inherent nature of ontologies. To bring ontology back to a normal state, we need to resolve these conflicts as given in Algorithm 2.

The proposed change management scheme provides up to date recommendations to the patients based on their recent activities performed and situation. After each change implementation, CM logs the changes in the Change Log and maintains these changes for later audit purposes including change recovery and for understanding the KR evolution history. A detailed Change Log structure was developed in ontology. The algorithms for CM and Rule-base evolution are given in Algorithms 2 and 3 respectively.

**Algorithm 2.** Change Management combined algorithm.***Algorithm ChangeManagement (Request, Change):***This algorithm has multiple sub-modules that are responsible for different subtasks like DAR or Rule Base Recovery. These are just called from the main algorithms.**Input:**
*Request* (change or audit or recovery) from user or system agent. Dynamic Activity ontology *O***Output:** Execution of request.1. /* Perform these steps if the request is a change request */2. **If**
*Request = “ChangeRequest”*
**then**3. /* Receive change request and formally represent the request */4.  *ChangeRequest* ←getChangeRequest(*Change*)5.  *ChangeRequest* ← *ChangeRequest* .formulate(*ChangeRequest,O*)6. /* Resolve syntactic and semantic conflicts because of change request */7.  *ChangeRequest* ← reform(*ChangeRequest* ,*O*)8. /* Implement the requested changes, propagate the changes and log the implemented changes */9.  *O* ← implement(*ChangeRequest*)*10.*  *propagate(ChangeRequest, RuleBase)**11.*  *logChangeRequest(ChangeRequest, ChangeLog)*12. **EndIf**13. /* Call recovery module if the request is for recovery */14. **If**
*Request = “ChangeRecovery”*
**then**   *recovery.execute(Change)*15. **EndIf**16. /* Call audit module if the request is for audit */17. **If**
*Request = “ChangeAudit”*
**then**   audit.execute(*Change*)18. **EndIf***End*

**Algorithm 3.** A Self Evolutionary Rule-base algorithm for updates in Rule DB to accommodate the evolving domain knowledge.***Algorithm EvolutionaryRule-base ( ):***Self Evolutionary algorithm to evolve Rule-base for accommodating the updated rules. Here *∆* represent a simple (unit level change) change whereas *RulesType* can be any type discussed in Figure 5. Expert rules and already existing rules are manipulated for possible evolution of rule in Rulebase.**Input:**Rules form Rule DB, newly generated rules from Inference Engine.**Input:**User entered rules for new knowledge.**Output:**Set of new and updated rules stored in Rule DB.**1.** /* Check for type of inference engine and then activate appropriate wrapper.*/**2.** *Wrapper.initiate(IE.RulesType)***3. **/* Fetch the rules generated by the inference engine or expert entered rules.*/**4.** *Rules ← IE.***5.** *Rules + ← EXPERT.Rules***6.** /* Fetch rules from RuleDB to be updated.*/**7.** *Rulesdb ← RuleDB.Rules***8.** **Loop**
*until Rules.next≠ null*
*a.* /* Check if rule from inference engine is new then add in RuleDB.*/*b.* **If***NOT(Rules.condition.next= ∃Rulesdb.condition.next)then**c.* *Rulesdb ← {x ∣ <Rules∆, x >New}* **d.** **Endif***e.* /* Check for rule updates, update rule, and store in RuleDB*/*f.* **If***(Rules.action.next= ∃RulesDB.action.next) ⊓ *(∃∆ ⊓ ∆.Rules.action.next.Change) ⊓ (Rules.next.support>RulesDB.next.support) then*g.* *Rulesdb.next ← {x ∣ <Rules∆, x > Change}* **h.** **Endif****9.** **EndLoop****10.** /* Update the original RuleDB for the new and updated rules.*/**11.** *Execute.update(RulesDB,Rulesdb)***End**

## 4. Implementation and Results

**Figure 8:**
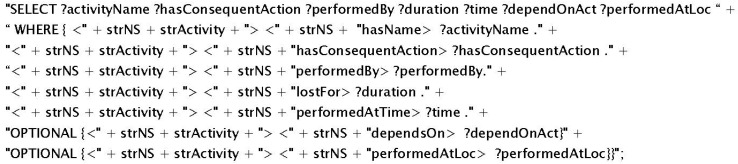
SPARQL query to extract information for match making.

The DDSS system is developed to especially support elderly patients in their daily life activities, nutrition intake, and social interaction to eliminate the problem of isolation and to help them learn from the experience of others. Smartphone has been used as the PA (main input source). Nine sensors (*i.e.*, GPS, Accelerometer, Camera, Proximity, Light, Time, Gyroscope, Bluetooth, and Microphone) and SMS service have been used to collect data (patient activity and experience information). All the experiments presented in this section are conducted on a local machine with 4 GB memory and 2.67 GHz Quad Core processor. To implement DDSS, Jena2, Protégé, Protégé-OWL, and Arq have been used. Accuracy of DDSS recommendations is dependent on the reported activities from the PA. For the inference module to work, the relevant information is extracted from KR using SPARQL queries (an instance is given in Figure 8). This query will extract related details for the activity, performer, and the duration from the KR for the purpose to be used in the inference/reasoning process.

Ontology matching system is used to support the match making process for newly detected activities against the existing knowledge from KR. For the selection of matching system, different ontology matching systems (*i.e.*, Falcon [28], Lily [29], and AgreementMaker [30]) have been tested on HL7 (Health Level–7) (two versions* i.e.*, HL7 version 2.5 and HL7 version 3.0) and openEHR (one version) ontologies for the system’s performance and accuracy. Falcon has produced 16 matches between HL7 version 2.5 and openEHR in 0.23 milliseconds whereas between HL7 version 3.0 and openEHR, 16 matches are found within 0.26 milliseconds. Lily on the other hand has produced 15 matches between HL7 version 2.5 and openEHR in 0.31 milliseconds whereas between HL7 version 3.0 and openEHR, 17 matches are found within 0.34 milliseconds. AgreementMaker has produced the best results both for the number of matches and for the time required. It has produced 16 matches between HL7 version 2.5 and openEHR in 0.13 milliseconds whereas between HL7 version 3.0 and openEHR, 18 matches are found within 0.15 milliseconds. Based on these results (in terms of accuracy and performance), AgreementMaker has been selected to be used for the match making process of the proposed system.

**Figure 9:**
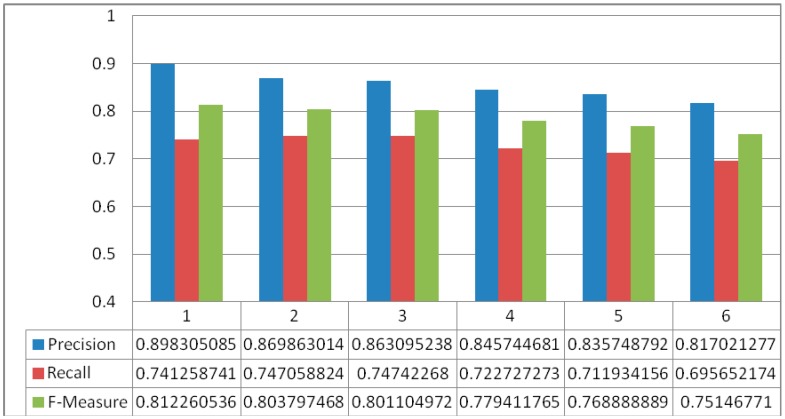
DDSS results in terms of precision, recall, and f-measure of match making process for 6 different experiments.

**Figure 10:**
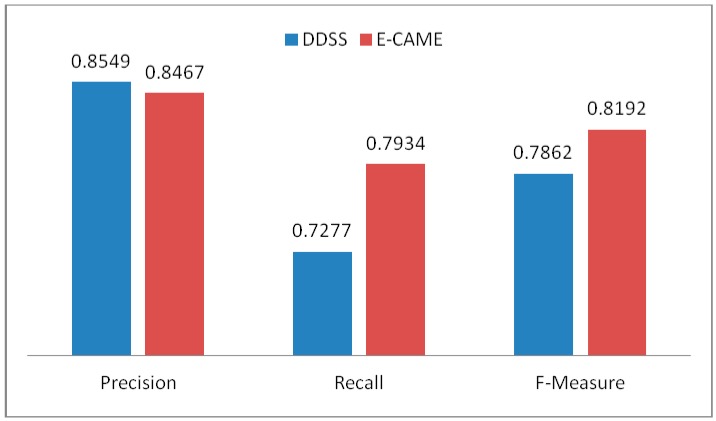
Average results in terms of Precision, Recall, and F-Measure of the proposed system and Extended CAME (E-CAME) [19] for a set of 36 experiments.

DDSS has been tested on the dataset used in [19,31] for a variety of different experiments with increasing number of activities. In each subsequent experiment, the number of activities increased as the user continuously interacted with the system and these activities are stored in the KR. Once an activity is reported by PA then it is forwarded to IE. For each activity the IE starts the reasoning and uses the information from KR. The information from KR is both the instance and structure information. The instance information is the actual information that is used in the match making process; however, the structure is used to limit the unintended activity (*i.e.*, unrecognizable activity which is not defined in set of recognized activities) information. In short, both T-Box (for structural reasoning of the knowledgebase) and A-Box (used for instance level reasoning of the knowledgebase) match making have been applied in a sequence. Figure 9 shows the results of match making process for DDSS where the y-axis represents the % of Precision, Recall, and F-Measure for the match making process while the x-axis represents the number of experiments. The data for knowledgebase is extracted from the activity set presented in [19] and also additional activities were recognized using [24]. This make the activity set independent and constraints free. The average precision, recall, and f-measure found in the set of six experiments are 0.8549, 0.7276, and 0.7862 respectively. The greater values for precision, recall and f-measure shows good results. However, with increase in data (number of activities set) affect the overall results. It is clear from Figure 9 that the accuracy is decreasing with the increasing number of activities. So the result of match making is not enough for recommendation generation. To validate the proposed system results, we have compared it with [19] that also consider to use both T-Box and A-Box reasoning processes followed by rule-based filtering. Both the systems were executed for a set of 36 experiments and the average Precision, Recall, and F-Measure of the experiments are given in Figure 10. The result of the match making phase has then been filtered with the rules from Rule-base to make appropriate personalized recommendations. Examples of rules are shown in Figure 5.

**Figure 11:**
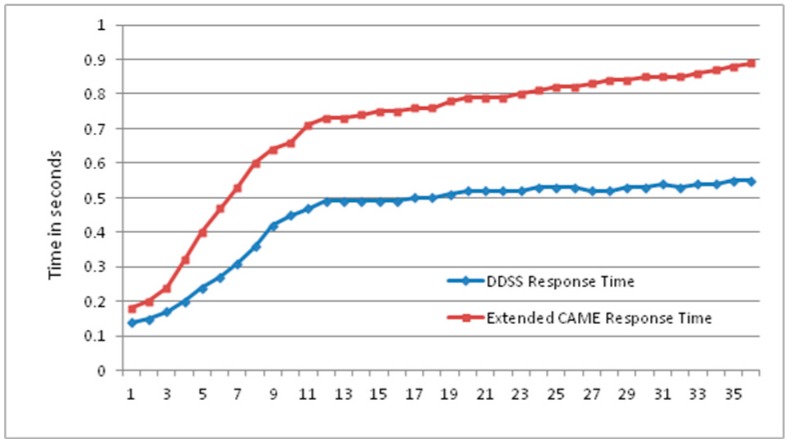
Response time comparison of proposed system recommendation generation against Extended CAME [19].

The Extended CAME [19] is a context-aware activity manipulation engine that will use sequence of activities and recognize their possible higher level activity. Due to its relevance, the proposed system is then compared against Extended CAME for the response time of both systems. As shown in Figure 11, the proposed system takes less time with an increasing number of activities. The proposed system has performed better than Extended CAME. However, the result main aim is to demonstrate the continuous increase of gap between the two systems’ with continuous increase of data. This eventually effect the overall response time of the compared systems. A greater number of activities will introduce more gaps between the response time of the proposed DDSS and Extended CAME. These results clearly demonstrate the performance advantage of the proposed system against Extended CAME.

**Figure 12:**
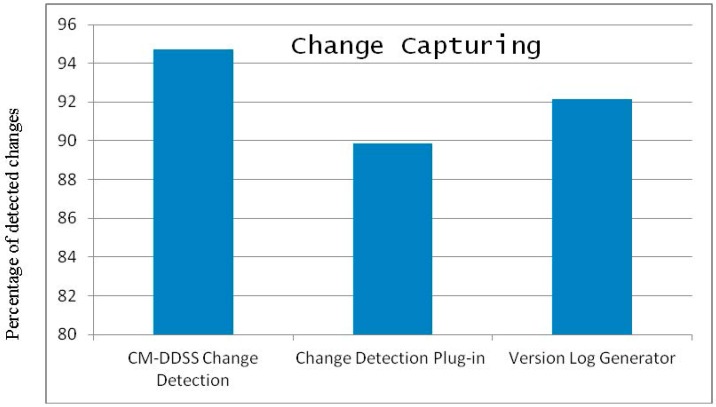
Change detection results of proposed CM of DDSS, *Change Detection Plug-in* and *Version Log Generator *[32].

The CM module has also been implemented and the result of change listening to the CM module is compared with existing systems* i.e.*, *Change Detection Plug-in* and *Version Log Generator* [32]. The system discussed in [32] is responsible detecting changes between two versions of ontologies by comparing them and identifying the changed resources. The proposed module shows better performance than the existing systems in terms of detecting and collecting the number of changes applied to otology. Figure 12 shows the results of the proposed component against the existing systems for a set of 20 experiments where each experiment consists of 35 random changes. Figure 12 show that the *Change Detection Plug-in *and *Version Log Generator *have detected 89.85% and 92.14% changes respectively. In comparison, our proposed CM of DDSS has detected 94.71% changes and has shown better performance than the existing systems. The reason for missing some of the changes are that Protégé does not trigger events for certain changes (*i.e.*, range deletion of Data Type property) and hence the change is not detected. For CM of DDSS to make complete recovery from one state of the KR ontology to another state depends on the detailed level logging of all the changes between the two states of the KR ontology. As shown in Figure 12 the proposed system has missed some changes and which actually are of critical importance. Any single change can bring inconsistency in the overall knowledge repository. Then this inconsistency will propagate further due to the associated and connected nature of resources in ontology. In addition, to provide accurate and robust recommendation services the change needs to be reflected efficiently, reliably and completely. To capture the information of these missed changes and log them for the purpose of achieving accurate recovery, the *diff()* function of the *Model* class from Jena API has been used. This gives the changed information between two models. So the missed changes have been recorded using *diff()* function where *diff() *function was applied after each single change event.

**Figure 13:**
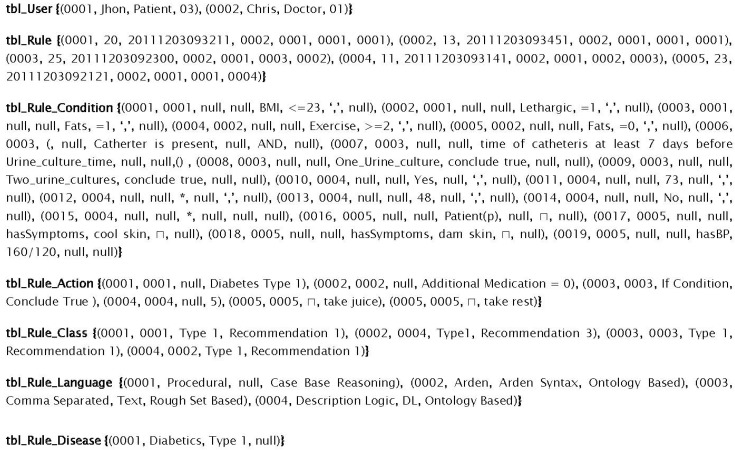
Shows the storage of rules given in Figure 2.

For DDSS, the Rule-base was developed and maintained in Microsoft SQL Server. Figure 13 shows the storage format of different rules given in Figure 5 using the proposed structure of Rule-base. All the attributes in conditions and actions are represented in columns, and are of fixed number. The values for the conditions are separated using commas that represent the AND operation. This generic structure for rules representation and storage mainly provides the facility to manipulate and dynamically update the rule-base. This is also independent of the rule language used for rules representation. The structure is important for the purpose to facilitate DDSS with reasoning engines of diverse nature rules and unbounded number of parameters which is one of the concerns of the existing reasoning systems. In Figure 13, seven relational tables are used to manage all relevant aspect of the rules concisely and decoupled. However, for testing of the proposed system, initially we started with 75 rules (in the rule set for testing the proposed system) based on different values for different combinations of symptoms. These are 33 in every single rule and these symptoms participate in each rule as the conditions of that rule. With the system learning new rules and experts suggesting new and updated rules, we have tested our system for its efficiency using the proposed structure with database manipulation against text files. We introduced a bunch of random 20 rules (*i.e.*, new rules, existing rules, and updated rules) and tested both technique’s performance. 

**Figure 14:**
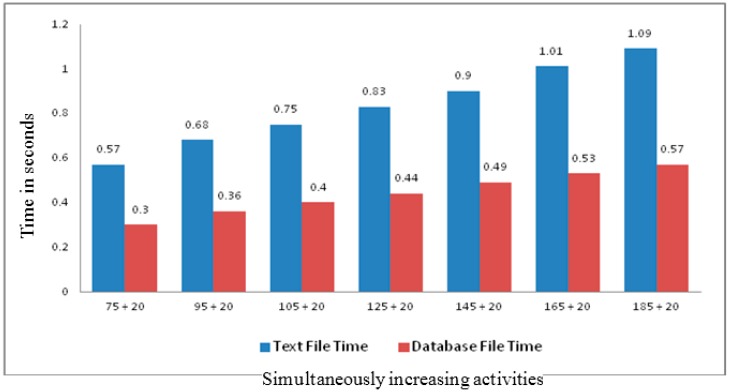
The performance comparison of proposed Self Evolutionary Rule-base against Text based rules storage.

The novelty of introducing dynamic evolution of Rule-base is to eliminate the need of change management by the human user which is time consuming and error prone. In addition, our proposed generic structure for rule storage also provides better performance than the traditional text file based rules storage. In Figure 14, the x-axis shows the number of experiments with existing rules in the Rule-base and the 20 additional rules. The y-axis represents the execution time in fraction of seconds required by the proposed algorithm. In the experiments, the first two sets of 20 rules are those generated by our proposed IE while the remaining five sets of 20 changes are user generated random changes for our proposed algorithm testing.

The dynamic evolution of Rule-base not only helps in timely updates in the Rule-base, in addition, the rules generated also become more compact. The proposed process is completely automatic. In addition, for generated rules verification the system also has provision for experts (doctors) to verify the rules and eliminate rules that are not compliant with standard knowledge of the domain.

Match making and rule based filtering generate appropriate recommendations to the patients for their daily nutrition intake, physical activities and social interactions. However, the results highly depend on the activity recognition results of the PA. Due to the continuous evolution of domain knowledge and dynamic user behavior, the CM continuously updates KR that helps DDSS to generate recent, and personalized services and recommendations.

## 5. Conclusions

Up to date user’s daily life information (*i.e.*, nutrition-related, physical activity-related and social interaction-related) collection and recommendation/services generation has been the focus of this research paper. With the constantly evolving domain knowledge and changing user behavior/experience, system adaption to the situation is very important. The proposed system read and learns from the environment and responds back with services and recommendations appropriately. The overall system results show encouraging accuracy and performance for recommendations generation, change management, and rule update. Better accuracy of personalized recommendation generation is the main future concern. In healthcare both performance and accuracy of the system are very important; however, as shown in the results, the system still needs optimization in terms of accuracy and response time.
